# ALGORITHMICALLY DEFINED SENSORIMOTOR FUNCTION AND RISK OF MILD COGNITIVE IMPAIRMENT

**DOI:** 10.1093/geroni/igae098.1196

**Published:** 2024-12-31

**Authors:** Amal Wanigatunga, Hang Wang, Anis Davoudi, Ryan Dougherty, Qu Tian, Eleanor Simonsick, Alden Gross, Jennifer Schrack

**Affiliations:** Johns Hopkins Bloomberg School of Public Health, Baltimore, Maryland, United States; Johns Hopkins University, Baltimore, Maryland, United States; Johns Hopkins University, North Bethesda, Maryland, United States; Rutgers University, Stoughton, Wisconsin, United States; National Institute on Aging, Baltimore, Maryland, United States; National Institute on Aging, Baltimore City 0400 Baltimore City, Maryland, United States; Johns Hopkins Bloomberg School of Public Health, Baltimore, Maryland, United States; Johns Hopkins Bloomberg School of Public Health, Baltimore, Maryland, United States

## Abstract

Early detection of persons at risk for, or living with, mild cognitive impairment (MCI) is a public health priority. Sensorimotor functioning—the integration of sensory information with motor function signaling to perform tasks and interact with the environment—may be a specific domain intertwined with movement planning and mental ability. However, a gap remains as to the optimal approach to operationalize sensorimotor function. This study assessed sensorimotor functioning using common clinical and research measures and evaluated its cross-sectional association with MCI. A unidimensional sensorimotor functioning factor was developed using confirmatory factor analysis based on six binary sensory and motor function impairment indicators including hearing, vision, olfaction, standing balance, usual gait speed, and grip strength. Using structural equation modeling, we examined whether higher sensorimotor functioning (greater factor score indicating less impairment) was related to lower odds of MCI using two independent cohort studies: The Atherosclerosis Risk and Communities Study (ARIC) (N=932), and the Baltimore Longitudinal Study of Aging (BLSA) (N=681) of participants who had complete sensory and motor function tests. After covariate adjustment, for each additional SD higher in sensorimotor factor score, there was a 56% (95%CI: 40%-68%) and 45% (95%CI: 24%-60%) lower odds of MCI in ARIC and BLSA, respectively. Findings suggest that leveraging a sensorimotor functioning score derived from common assessments is feasible and associated with MCI. Longitudinal studies are needed to determine whether degrading sensorimotor function can predict early cognitive decline indicative of elevated dementia risk.

